# Evaluation of Atmospheric Effects on Interferograms Using DEM Errors of Fixed Ground Points

**DOI:** 10.3390/s18072336

**Published:** 2018-07-18

**Authors:** Takashi Nonaka, Tomohito Asaka, Keishi Iwashita

**Affiliations:** College of Industrial Technology, Nihon University, Chiba 2758575, Japan; asaka.tomohito@nihon-u.ac.jp (T.A.); iwashita.keishi@nihon-u.ac.jp (K.I.)

**Keywords:** synthetic aperture radar, interferogram, digital elevation model, single-pass

## Abstract

High-resolution synthetic aperture radar (SAR) data are widely used for disaster monitoring. To extract damaged areas automatically, it is essential to understand the relationships among the sensor specifications, acquisition conditions, and land cover. Our previous studies developed a method for estimating the phase noise of interferograms using several pairs of TerraSAR-X series (TerraSAR-X and TanDEM-X) datasets. Atmospheric disturbance data are also necessary to interpret the interferograms; therefore, the purpose of this study is to estimate the atmospheric effects by focusing on the difference in digital elevation model (DEM) errors between repeat-pass (two interferometric SAR images acquired at different times) and single-pass (two interferometric SAR images acquired simultaneously) interferometry. Single-pass DEM errors are reduced due to the lack of temporal decorrelation and atmospheric disturbances. At a study site in the city of Tsukuba, a quantitative analysis of DEM errors at fixed ground objects shows that the atmospheric effects are estimated to contribute 75% to 80% of the total phase noise in interferograms.

## 1. Introduction

Recently, commercial high-resolution synthetic aperture radar (SAR) satellites, such as TerraSAR-X [1] (developed by the German Space Center (DLR)), COSMO-SkyMed [2] (developed by the Italian Space Agency), and Radarsat-2 [3] (developed by the Canadian Space Agency), have been widely used for significant applications, such as in the monitoring of natural hazards and disasters [4]: Satellite data are useful for acquiring the scale and center of damage in a huge disaster. Images of both before and after such events, are used to acquire the damaged area in general. For example, a flooded area was estimated by utilizing a feature wherein the backscattering coefficients decreased after the flooding [5]. Damaged man-made buildings were extracted, using a feature wherein the coherence (i.e., the amplitude of the complex correlation coefficient between two SAR images) decreased for objects that underwent change [6]. In addition, small displacements were estimated by differential interferometry [7] and pixel matching [8]. However, those studies set the processing parameters of the method and determined the threshold according to the situation. The errors in a differential interferogram depend on the acquisition conditions (e.g., baseline length and acquisition interval) and the sensor specifications (e.g., wavelength and resolution). In addition, temporal decorrelation in vegetation areas and atmospheric disturbances (ADs) exhibit inherent accuracy limitations [9,10]. Therefore, these factors inhibit the automation of these methods to access any disaster and impede the process of providing information to end users without time delays. As such, guidelines are required to determine the parameters and thresholds required to use satellite data effectively. 

In order to construct a methodology for tackling any situation, it is essential to acquire comprehensive knowledge of both the basic features of the sensors and the acquisition conditions from the various datasets. To clarify the coherence variation that is due to the baseline and acquisition interval, several studies revealed that the coherence produced under various acquisition conditions differed, according to the land cover that was being assessed; namely, the difference between man-made and natural objects [11]. Other studies evaluated the relative geolocation accuracy through the use of many pairs of TerraSAR-X data to understand the displacement errors estimated by pixel matching [12]. Recently, we focused on interferometric noise (noise produced by many types of acquisition-condition datasets in previous studies [13]), as a useful tool to evaluate the displacement errors derived from differential interferogram analysis. We proposed a method for estimating interferometric noise using models that consider the ratio of digital elevation model (DEM) errors to the height of ambiguity (HoA). We then estimated the noise of TerraSAR-X, Advanced Land Observing Satellite (ALOS) PALSAR, and ALOS-2 PALSAR-2 by repeat-pass observations [13,14,15]. However, two interferometric SAR images are not simultaneously observed in the case of repeat-pass observation, and the radiation propagation for each of the images can be affected differently by the atmosphere. In particular, different atmospheric humidity, temperature, and pressure between the two acquisitions will exhibit a visible effect on the interferometric phase. Furthermore, the target in each resolution cell may be altered during the time interval between two SAR acquisitions. Therefore, repeat-pass interferograms include errors that are caused due to ADs and temporal variations in vegetation [10]. Currently, single-pass observations have been conducted using both the TerraSAR-X and TanDEM-X satellites. Single-pass observations can produce DEMs with high accuracy because ADs and the temporal-variation component are eliminated by simultaneous observations by TerraSAR-X and TanDEM-X. The difference between single-pass and repeat-pass corresponds to these ADs and the temporal-variation components. Therefore, the purpose of the present study is to estimate the effects of ADs and the temporal variations of objects using both single-pass and repeat-pass observations. First, we explain the method developed to estimate interferometric noise by analyzing DEM errors for fixed ground points (e.g., roads, parking lots, and parks). We then examine both the single-pass and the repeat-pass imaging DEM errors for a case study in the city of Tsukuba, Japan. Finally, we evaluate the interferometric noise caused by ADs and temporal decorrelation. 

## 2. Data and Study Site

### 2.1. Data

#### 2.1.1. TerraSAR-X/TanDEM-X

Launched in 2007, TerraSAR-X is an advanced SAR satellite system for scientific and commercial applications, which was realized in a public–private partnership between DLR and Astrium GmbH. It carries X-band (9.65 GHz) SAR sensors and operates in several imaging modes [16]: The 300-MHz high-resolution Staring Spotlight mode achieves a spatial resolution of 25 cm (azimuth); the StripMap and ScanSAR modes allow acquisition with resolutions of 3 and 18 m, respectively; the Wide ScanSAR mode covers a swath width of 200–270 km depending on the incidence angle. Table 1 lists the orbit parameters [17]. A sun-synchronous circular dawn–dusk orbit is adopted with a local time of the ascending node at 1800 h (±0.25 h) of equatorial crossing, an average altitude of 514.8 km (505–533 km), an inclination of 97.44°, and a nominal revisit period of 11 d (167 orbits within one revisit period, at a rate of 15 2/11 orbits per day).

Launched in 2010, TanDEM-X (TerraSAR-X’s add-on for DEM) is a radar satellite that is almost identical to TerraSAR-X [18]. It revolves around the Earth together with TerraSAR-X as a unique satellite formation. As the first bistatic (BS) SAR system in space, TanDEM-X’s main goal is to generate a global DEM, following the HRTI-3 standard [19]. Operational DEM generation is planned to be performed using BS interferometry (BS mode), which is characterized by the illumination of a scene by one transmitter and the simultaneous measurement of the same scene with two receivers, thereby avoiding temporal decorrelation [20]. 

The present study uses several pairs of BS-mode data of TerraSAR-X/TanDEM-X as well as the StripMap (SM) and High-resolution SpotLight (HS) mode of TerraSAR-X (Table 2). The three BS pairs are used for single-pass interferometry, and six SM images and five HS images are used for repeat-pass interferometry. The interval for acquisition is roughly 4 y for these data. The incidence angle of each mode is almost same, but the polarizations and orbit directions are different.

#### 2.1.2. Panchromatic Remote Sensing Instrument for Stereo Mapping DEM

Panchromatic Remote Sensing Instrument for Stereo Mapping (PRISM) was one of the sensors used on the ALOS, which operated from 2006 to 2011 [21]. PRISM was comprised of three panchromatic radiometers that acquired along-track stereo images. It had a spatial resolution of 2.5 m in its nadir-looking radiometer and achieved global coverage, making it suitable as a potential candidate for a precise global DEM. The reference DEM used in the present study is the “ALOS World 3D” dataset generated by PRISM. The project was conducted by the Japan Aerospace Exploration Agency in collaboration with commercial partners NTT DATA Corp. and the Remote Sensing Technology Centre of Japan (RESTEC). It comprises a precise DEM in 1° tiles of lat/long frame units, and ortho-rectified images of the nadir in scene bases. The horizontal resolution of the mesh was approximately 30 m (1 arcsec), and the target height accuracy was 5 m, as measured by the root-mean-square error (RMSE) [22]. We selected the validation points from the flat terrain and confirmed that the accuracy of the PRISM DEM was 2 m in comparison with that observed in the reference data. The reference data represented the airborne data. The accuracy of the reference data was ~10 cm in comparison with that of in situ data.

### 2.2. Study Site and Validation Points

**Figure 1:**
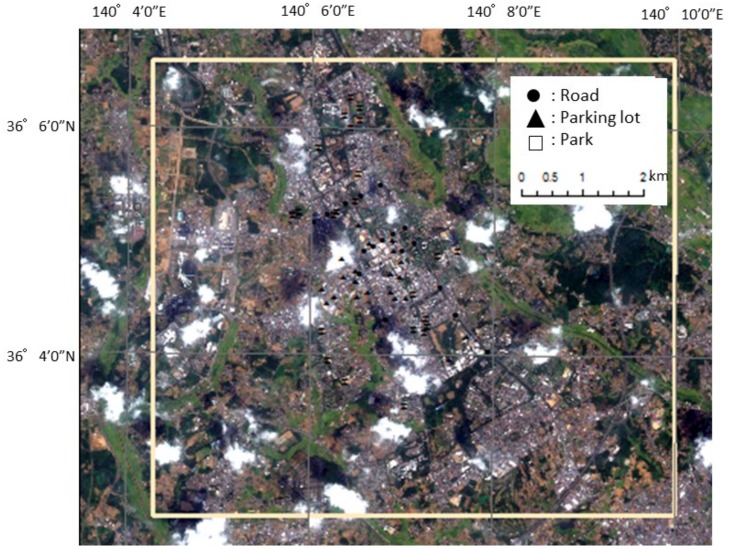
Study site (yellow box) of Tsukuba City in Ibaraki Prefecture and digital elevation model (DEM) validation points (roads, parking lots, and parks) produced from TerraSAR-X data.

The study site was located in the city of Tsukuba in the southern part of Ibaraki Prefecture and measures roughly 64 km^2^ (8.5 km × 7.5 km; Figure 1). The flat topography of 20–30-m elevation was covered by the loamy layer of the Kanto Region. Tsukuba has undergone rapid development, owing to recent urbanization. High-rise apartments have now been constructed around the Tsukuba station at the center of the study site. The atmosphere over the small, flat site was assumed to be spatially homogeneous to a certain extent. Man-made fixed objects are suitable to be selected as validation points, but the elevation of tall buildings is inaccurate because of the associated phase unwrapping difficulty, thereby yielding an inaccurate DEM. Therefore, the validation points were selected from areas of fixed objects with a flat terrain (Figure 1). The validation points used included15 roads, 18 parking lots, and 14 ground objects (points with large error deviations were removed from the analysis to evaluate the noise [23]). The validation points can be recognize using the PRISM DEM. In addition, the DEM errors caused by temporal variation were small for these objects, making them suitable for evaluating errors due to atmospheric effects. 

## 3. Method

SAR interferometry (InSAR) allows topography to be measured with metric accuracy by exploiting the phase difference between the backscattered microwave signals of two images that were received from slightly different positions [24,25]. If the two SAR acquisitions are also displaced by a time interval, ground displacements that occurred between the two acquisitions can be detected with millimetric precision in a technique known as differential InSAR (DInSAR) [26]. The phase (*φ*) is expressed as [10]:

(1) $$\phi =\varphi +\frac{4\pi }{\lambda }r+\alpha +\nu ,$$

where *φ* is the reflectivity of the radar target, *4πr/λ* is the propagator depending on the sensor–target distance, α is the atmospheric phase contribution, and *ν* is related to noise (thermal noise is most important). The interferogram phase (*dϕ*) is:

(2) $$d\phi =\phi_{flat}+\phi_{elevation}+\phi_{displacement}+\phi_{atmosphere}+\phi_{noise},$$

where *ϕ_flat_* is the phase contribution of the flat earth, *ϕ_elevation_* is that of the elevation, *ϕ_displacement_* is that of the deformation, *ϕ_atmosphere_* is that of the atmosphere, and *ϕ_noise_* is that of the noise. Because two interferometric SAR images are not observed simultaneously in the case of repeat-pass interferometry, the radiation travel path for each can be differently affected by the atmosphere. Therefore, *ϕ_atmosphere_* will exhibit a visible effect on *dϕ* in case of repeat-pass interferometry, whereas it will barely exhibit any effect on *dϕ* in the case of single-pass interferometry because the simultaneous acquisition negligibly affects the travel path.

The Single-look Slant-range Complex (SSC) product was used for repeat-pass interferometry. SSC is a single-look product of the focused radar signal. The pixels are spaced equidistant in the azimuth (according to the pulse repetition interval (PRI) = 1/pulse repetition frequency (PRF)) and slant range (according to the range sampling frequency). The data are represented as complex numbers containing amplitude and phase information. Each image pixel is processed to zero Doppler coordinates in the range direction. For single-pass interferometry, the Coregistered SSC (CoSSC) product was used instead. This product was generated by a nominal processing chain and was mainly provided as an output of the intermediate SSC files generated after focusing and coregistration [27].

**Figure 2:**
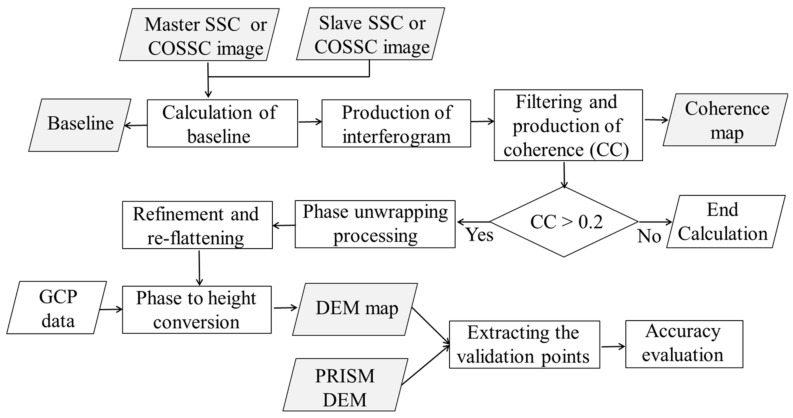
Flowchart of DEM map production by interferometry for both repeat-pass and single-pass interferometry along with height accuracy evaluation by comparing with Panchromatic Remote Sensing Instrument for Stereo Mapping (PRISM) DEM.

The procedure for DEM generation is depicted in Figure 2. First, two images (called master and slave) were coregistered, using the DEM to align the same points in the two images, and the perpendicular baseline was estimated from the orbit ephemerides. Then, an interferogram [number of looks: 5 (azimuth) × 4 (range) for SM and 5 × 3 for HS] was generated after spectral shift filtering was performed on the image pair. Synthetic fringes were generated from a coarse-resolution SRTM DEM using a backward geocoding approach and were then cross-multiplied by the SAR interferogram in the next step. This step allowed the removal of most of the low-frequency components of the wrapped phase to ease the following phase unwrapping. After that, a Goldstein filter was used to filter the interferogram phase [28] because it significantly improves fringe visibility and reduces the noise introduced by temporal- or baseline-related decorrelation, and the corresponding coherence was subsequently generated. The interferogram phase was further unwrapped using the Delaunay Minimum Cost Flow method [29] and an unwrapping coherence threshold of 0.2 (pixels with coherence values smaller than this threshold were not used for unwrapping). As a result, only the points with good coherence were unwrapped, without any influence from the low-coherence pixels. In the next step, the refinement and re-flattening were performed using SRTM DEM, which is an important step for accurately transforming the unwrapped phase information into height values. It allows both refinement of the orbits (i.e., correcting possible inaccuracies) and the calculation of the phase offset (i.e., obtaining the absolute phase values). In the final step, the absolute calibrated and unwrapped phase was recombined with the synthetic phase of SRTM DEM and then converted to height. Geocoding was performed using the most commonly adopted Range-Doppler method [30]. The pixel size for SM and BS was 10 m, and that for HS was 5 m. To evaluate the DEM noise, the height of each validation point was extracted from each image, a comparison was conducted between TerraSAR-X and the reference PRISM, and the RMS DEM errors were calculated.

## 4. Results

### 4.1. Baseline of Image Pairs

The height of ambiguity is defined as the height difference that generates an interferometric phase change of 2π after interferogram flattening. The height of ambiguity is inversely proportional to the perpendicular baseline:

(3) $$\begin{array}{cc} \frac{dh}{d\phi }=\frac{\lambda Rsin\theta }{2Bp} & \;\;\;\;\;\left(repeat-pass\right),\\ \frac{dh}{d\phi }=\frac{\lambda Rsin\theta }{Bp} & \;\;\;\;\;\left(single-pass\right), \end{array}$$

where *h* is the height, *φ* is the phase, *λ* is the wavelength, *R* is the slant-range length, *θ* is the incidence angle, and *B_P_* is the perpendicular baseline. The equations differ for repeat- and single-pass interferograms because the travel path difference for the repeat-pass interferogram is the twice that for the single-pass interferogram. In the TerraSAR-X/TanDEM-X case with *λ* = 3 cm, *θ* = 42°, and *R* = 690 km, the following expression holds (in m):

(4) $$\begin{array}{cc} \frac{dh}{d\phi }\approx \frac{6900}{Bp} & \;\;\;\;\left(repeat-pass\right),\\ \frac{dh}{d\phi }\approx \frac{13800}{Bp} & \;\;\;\;\;\left(single-pass\right). \end{array}$$

In principle, the longer the baseline, the more accurate the height measurement because the phase noise is equivalent to smaller DEM errors. However, an upper limit exists for the perpendicular baseline, over which the interferometric signals decorrelate and no fringes can be generated. Therefore, for high-quality repeat-pass SAR interferometry, the satellite orbit must be well-controlled. In the case of TerraSAR-X, the motion of TerraSAR-X was controlled in a predefined tube of 250-m radius throughout the entire mission [31], making it possible to acquire a high-quality DEM by repeat-pass interferometry.

**Figure 3:**
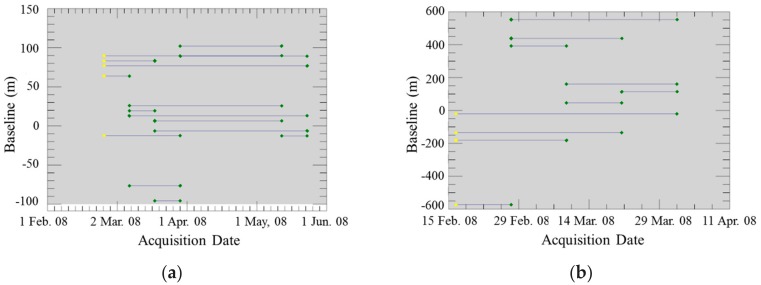
Comparison of the baseline for the repeat-pass interferometry of StripMap (SM) (**a**) and High-resolution SpotLight (HS) (**b**).The horizontal axis represents the acquisition date of the images.

Figure 3 shows each baseline pair for repeat passes of SM and HS. The maximum baseline was approximately 600 m. However, the combination with a large baseline included an image from 27 February 2008, for HS, and except for the pairs with this image, the baseline was less than 200 m. The average baseline for all pairs of data was 140 m, and its deviation was 160 m. The mean height of ambiguity that was calculated using all of these baseline values was approximately 200 m.

### 4.2. DEM Errors

**Figure 4:**
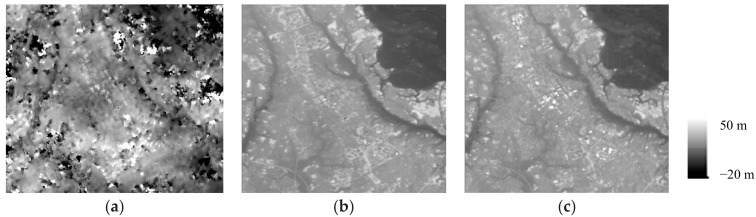
Comparison of the DEMs produced by (**a**) repeat-pass (26 February, 2008, and 24 May, 2008), (**b**) single-pass interferometry, and (**c**) PRISM DEM.

Figure 4 compares the TerraSAR-X DEM, the TanDEM-X DEM, and the reference PRISM DEM. Approximately the same features were found in both the TerraSAR-X and TanDEM-X DEMs, regardless of the selection of the acquisition mode and image pairs. The TerraSAR-X DEM was noisy, and the noise seemed to be larger because its baseline was shorter. The noise originated mainly from ADs and the temporal changes of natural objects. In contrast, TanDEM-X did not have such noise. The characteristics of up and down and the features of Tsukuba were well represented compared with the reference PRISM DEM. In addition, the high-rise buildings in the city center were underestimated [32]. This was caused by the difficulty of phase unwrapping the spatial discontinuous height changes. However, even though the targets were flat, the water surface (the pond inside the park) exhibited some height deviations. Another study showed that the single-pass DEM RMS errors were almost the same (deviation: 0.2 m) independent of the object categories (“road,” “parking lot,” and “park”) in the same pairs [23]. These studies indicated that these three types of fixed ground objects were suitable for evaluating the noise. 

### 4.3. Interferometric Noise

The model for estimating the interferometric noise is shown in Equation (5), which expresses the ratio of DEM error (DEM_ERR_) to the height of the ambiguity (dh/dφ):

(5) $$DEM_{ERR}=\alpha \frac{dh}{d\phi }+\beta ,$$

α represents the noise. α is expressed as 0.095 in Equation (6), which shows the equation of the linear regression curve that was obtained using least-squares fitting to the DEM errors in the observed data and the height of ambiguity calculated by Equation (3):

(6) $$DEM_{ERR}=0.095\frac{dh}{d\phi }+1.4.$$

**Figure 5:**
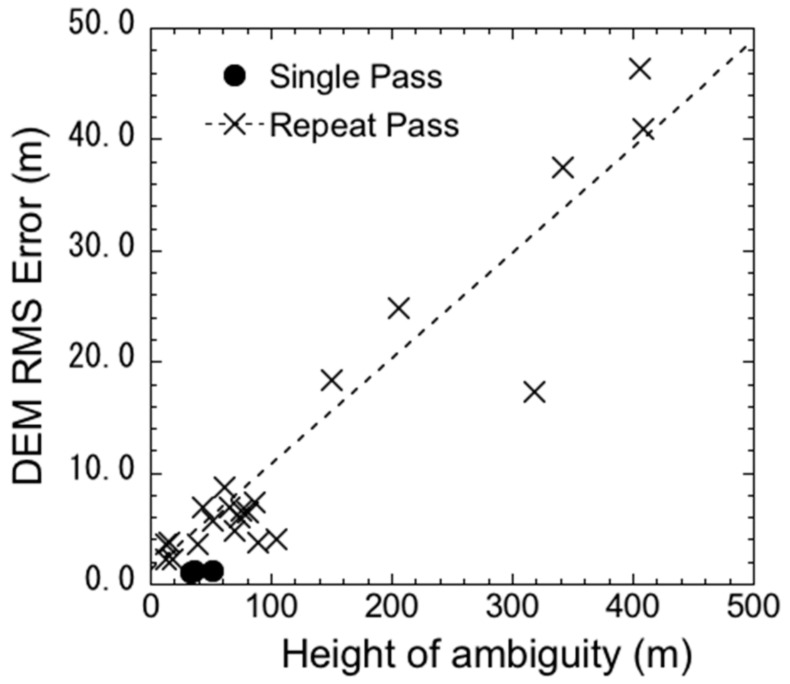
Comparison of the relation between DEM errors and height of ambiguity between repeat-pass and single-pass interferometry.

This indicates that the interferometric noise was 9.5% and that there was a significant (1%) relation between them, proving the validity of the method using fixed ground points. In addition, the estimated regression noise (9.5%) was almost the same for SM and HS [33]. Therefore, it was independent of the acquisition mode, baseline, and product pixel size. In addition, the fact that the relationship between them was independent of the acquisition interval implied that no temporal decorrelation was found in the DEM errors for these objects. Figure 5 compares the DEM errors of the validation points between single- and repeat-pass interferometry. Although only three pairs of data were acquired for single-pass interferometry, the DEM errors were only 1.1–1.2 m for a height of ambiguity of 30–50 m. These errors are 20–25% of those encountered for the same height of ambiguity for repeat-pass interferometry. This difference in the errors (75–80%) is considered to correspond to ADs.

### 4.4. Discussion

**Figure 6:**
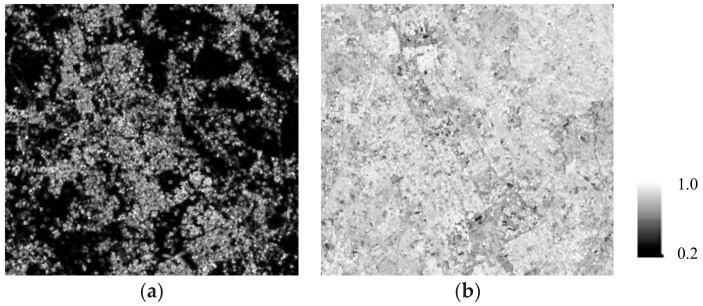
Comparison of coherence produced by (**a**) repeat-pass (30 March and 13 May, 2008) and (**b**) single-pass (6 September, 2011) interferometry.

**Figure 7:**
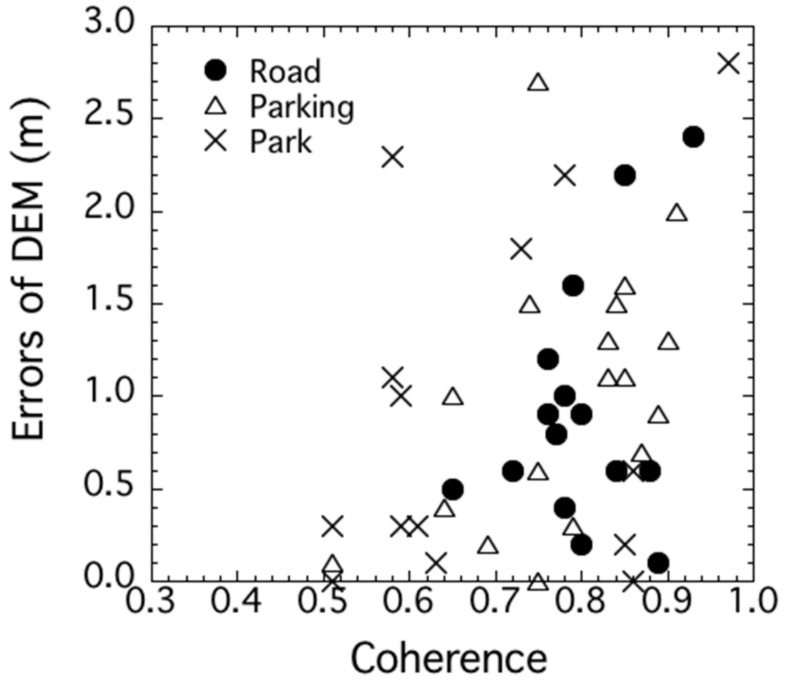
Comparison of the relations between DEM error and coherence for single-pass interferometry.

In the previous section, evaluating the differences among the DEM errors for the fixed ground points revealed the noise due to ADs. The decreased coherence for repeat-pass interferometry was one of the DEM error factors. Therefore, this section compares the coherence maps between repeat-pass and single-pass interferometry, and we reveal the coherence variation due to temporal decorrelation and ADs. Figure 6 and Table 3 compare the coherence maps and the statistical values, respectively. A comparison was made for pairs that exhibited similar heights of ambiguity. The average value of 0.82 for single-pass interferometry is twice the value of 0.41 for repeat-pass interferometry, and the maps clearly show that the increased values for natural objects (apart from those in the city center) contributed greatly. Without the effects of temporal decorrelation and ADs, a fairly small standard deviation of 0.1 was observed for single-pass interferometry. Actually, 75% of the values for single-pass interferometry exceeded 0.75, although several water bodies and pieces of bare ground exhibited a somewhat lower coherence of less than 0.5. Table 4 compares the coherence values of the fixed ground points for repeat-pass and single-pass interferometry. The average coherence for single-pass interferometry was 0.76, which was a little lower than the scene average of 0.82 (Table 3) and 0.27 higher than that for repeat-pass. Several of the park validation points had values lower than 0.5 despite the lack of temporal decorrelation for single-pass interferometry. In contrast, the roads and parking lots had high coherence values for all the points, and these were stable points for interferometry. Figure 7 shows the relationships between the DEM errors and the coherence for single-pass interferometry. The results show that the higher the coherence, the more the deviation of DEM errors except for parks. This result can be explained by the fact that pairs with short baseline tend to exhibit high coherence as well as large height of ambiguity, which results in more amount of DEM errors (Equation (5)). Therefore, we conclude that small differences in coherence affect the DEM accuracy, even for single-pass interferometry. This study dealt with only three pairs of data for single-pass interferometry based on the restriction that was placed on the number of BS acquisitions by both TerraSAR-X and TanDEM-X. In a future work, we will analyze the single-pass interferometric noise more accurately using the relations between RMS DEM errors and the height of ambiguity based on data with various baseline lengths.

## 5. Conclusions

Differential interferometry is extensively used to acquire ground displacement at the centimeter level. However, a problem is that the interferograms contain errors caused by ADs; thus, it was necessary to comprehensively understand these errors. Therefore, the purpose of this study was to evaluate the phase errors caused by ADs. We focused on the single-pass interferograms derived without ADs and compared them with the repeat-pass interferograms. In addition, the validation points were selected from fixed ground points, making it possible to remove the effects of temporal decorrelation. The results showed that the repeat-pass interferogram phase noise was roughly 9.5% and that the phase noise of single-pass interferogram was roughly 20–25% of that of the repeat-pass interferogram phase noise. This implied that 75–80% of the phase noise was caused by ADs and temporal decorrelation. We also compared the coherence, whose standard deviation was only 0.1 for single-pass interferometry for various land cover conditions without ADs and temporal decorrelation. The results also revealed that the deviation of DEM errors increased as the coherence increased, even for single-pass observation. In this study, the number of acquisition included only three pairs of single-pass observations. In a future work, we will analyze the single-pass interferometric noise more accurately, using the relations between RMS DEM error and height of ambiguity based on data with various baseline lengths. In addition, the present case study was limited to the site in Tsukuba and the estimated values depended on the local atmospheric conditions. Therefore, we need to apply the proposed method to data from other sites to increase the statistical reliability of our results in future work. 

## Figures and Tables

**Table 1:** TerraSAR-X orbit characteristics and orbit elements.

Orbit type	Sun-synchronous repeat orbit
Satellite altitude	505–533 km
Repeat period	11 d
Repeat cycle	167 orbits in one repetition
Local time of ascending node	1800 ± 0.25 h

**Table 2:** TerraSAR-X and TanDEM-X images used in this study.

Mode	StripMap	High-Res. SpotLight	Bistatic
Satellite	TerraSAR-X	TerraSAR-X	TerraSAR-X and TanDEM-X
Acquisition date	2008: 26 February, 8 March19 March, 30 March13 May, 24 May	2008: 16 February, 27 February9 March, 20 March31 March	2011: 6 September2012: 3 September, 6 October
Resolution (az, rg)	3.3 m, 2.7 m	1.1 m, 1.8 m	3.3 m, 2.6 m
Polarization	VV	VV	HH
Incidence angle	41.4°	42.1°	43.2°
Direction	Descending	Ascending	Ascending

**Table 3:** Coherence statistics (all coherence values in the study site were used for the analysis).

	Repeat-Pass Interferometry	Single-Pass Interferometry
Average	0.41	0.82
Standard deviation	0.18	0.09
1st quartile	0.25	0.77
2nd quartile	0.36	0.84
3rd quartile	0.54	0.88

**Table 4:** Comparison of the coherence values of the fixed ground points for repeat-pass (30 March and 13 May, 2008) and single-pass (6 September, 2011) interferometry according to object type.

	Repeat-Pass Interferometry	Single-Pass Interferometry
Road Parking lots Park	0.57 ± 0.13 0.47 ± 0.16 0.43 ± 0.15	0.80 ± 0.07 0.78 ± 0.11 0.69 ± 0.15
All fixed points	0.49 ± 0.16	0.76 ± 0.13
